# Inhibition of Notch Signaling Stimulates Osteoclastogenesis From the Common Trilineage Progenitor Under Inflammatory Conditions

**DOI:** 10.3389/fimmu.2022.902947

**Published:** 2022-07-05

**Authors:** Maša Filipović, Darja Flegar, Alan Šućur, Dino Šisl, Inga Kavazović, Mariastefania Antica, Tomislav Kelava, Nataša Kovačić, Danka Grčević

**Affiliations:** ^1^Department of Physiology and Immunology, University of Zagreb School of Medicine, Zagreb, Croatia; ^2^Laboratory for Molecular Immunology, Croatian Institute for Brain Research, University of Zagreb School of Medicine, Zagreb, Croatia; ^3^Department of Histology and Embryology, Faculty of Medicine, University of Rijeka, Rijeka, Croatia; ^4^Division of Molecular Biology, Ruđer Bošković Institute, Zagreb, Croatia; ^5^Department of Anatomy, University of Zagreb School of Medicine, Zagreb, Croatia

**Keywords:** myeloid progenitor, inflammation, Notch, osteoclast, macrophage, dendritic cell

## Abstract

Osteoclasts, macrophages and dendritic cells (DCs) can be derived from a common trilineage myeloid progenitor of hematopoietic origin. Progenitor commitment is susceptible to regulation through Notch signaling. Our aim was to determine the effects of Notch modulation on trilineage progenitor commitment and functional properties of differentiated cells under inflammatory conditions. We used the conditional inducible CX3CR1CreERT2 mouse strain to achieve overexpression of the Notch 1 intracellular domain (NICD1) or to inhibit Notch signaling *via* deletion of the transcription factor RBP-J in a bone marrow population, used as a source of the trilineage progenitor (CD45^+^Ly6G^−^CD3^−^B220^−^NK1.1^−^CD11b^–/lo^CD115^+^). Cre-recombinase, under the control of the CX3CR1 promoter, expressed in the monocyte/macrophage lineage, was induced *in vitro* by 4-hydroxytamoxifen. Differentiation of osteoclasts was induced by M-CSF/RANKL; macrophages by M-CSF; DCs by IL-4/GM-CSF, and inflammation by LPS. Functionally, DCs were tested for the ability to process and present antigen, macrophages to phagocytose *E. coli* particles, and osteoclasts to resorb bone and express tartrate-resistant acid phosphatase (TRAP). We found that Notch 1 signal activation suppressed osteoclast formation, whereas disruption of the Notch canonical pathway enhanced osteoclastogenesis, resulting in a higher number and size of osteoclasts. RANK protein and *Ctsk* gene expression were upregulated in osteoclastogenic cultures from RBP-J^+^ mice, with the opposing results in NICD1^+^ mice. Notch modulation did not affect the number of *in vitro* differentiated macrophages and DCs. However, RBP-J deletion stimulated *Il12b* and *Cd86* expression in macrophages and DCs, respectively. Functional assays under inflammatory conditions confirmed that Notch silencing amplifies TRAP expression by osteoclasts, whereas the enhanced phagocytosis by macrophages was observed in both NICD1^+^ and RBP-J^+^ strains. Finally, antigen presentation by LPS-stimulated DCs was significantly downregulated with NICD1 overexpression. This experimental setting allowed us to define a cell-autonomous response to Notch signaling at the trilineage progenitor stage. Although Notch signaling modulation affected the activity of all three lineages, the major effect was observed in osteoclasts, resulting in enhanced differentiation and function with inhibition of canonical Notch signaling. Our results indicate that Notch signaling participates as the negative regulator of osteoclast activity during inflammation, which may be relevant in immune and bone diseases.

## Introduction

Osteoclasts, macrophages and dendritic cells can be derived from the common trilineage myeloid progenitor of hematopoietic origin (1–4). Although developmentally related, these three cell lineages exhibit different and specialized functions in the bone marrow and peripheral tissues. Dendritic cells and macrophages are part of the mononuclear phagocyte system involved in tissue maintenance, innate immunity and pathogen clearance, as well as in the induction of adaptive immune responses (5). Macrophages are tissue scavenging phagocytic cells that internalize cell debris and bacteria. In addition, they produce inflammatory mediators, regulate tissue repair and participate in cellular immunity (6, 7). Dendritic cells are also phagocytic cells with the ability to induce an inflammatory reaction and process antigens for the initiation of the adaptive immune response (8, 9). Osteoclasts are unique bone-resorbing multinuclear cells, essential for the development and continuous remodeling of the skeleton and the bone marrow hematopoietic niche (10, 11). They are also involved in fracture repair and pathological bone resorption associated with inflammatory conditions (12, 13).

Several groups have identified and characterized a bone marrow-derived common progenitor for these three related cell types using flow cytometry combined with differentiation assays (1, 4). Investigations of murine bone marrow have identified immature myeloid CD117^+^CD115^+^RANK^−^ cells as progenitors with the ability of *in vitro* differentiation into osteoclasts and dendritic cells (14). It was also reported that the cell population with the phenotype Lin^−^CD115^+^CD135^+^CX3CR1^+^ corresponds to a progenitor subset with the ability to generate macrophages and dendritic cells (15). Further dissection revealed a rare (0.1–0.3%) B220^−^CD3^−^CD11b^–/lo^CD115^+^CD117^+^CX3CR1^+^ population as a common bone marrow progenitor pool for osteoclasts, macrophages and dendritic cells at the single-cell clonal level, expressing, to a certain degree, other myeloid markers such as Ly6C and F4/80 (2, 16, 17). By using a different combination of surface markers, Xiao et al. identified B220^−^CD117^+^CD115^+^CD11b^lo^CD27^hi^ subset as a common mouse bone marrow macrophage/osteoclast/dendritic cell progenitor (MODP) (18). Using the methodology and cues from the mouse study, the same group further reported existence of the equivalent MODP (CD11b^−^CD34^+^c-KIT^+^FLT3^+^IL3Rα^hi^) in human hematopoiesis (19). In addition, recent studies proposed that the expression of chemokine receptors, such as CCR2 and CX3CR1, in progenitor subsets is associated with pathological processes (13, 20, 21). In spite of a subtle heterogeneity of the phenotype profile reported by different studies, this identified and isolated progenitor retained the plasticity to differentiate into osteoclasts, macrophages and dendritic cells under appropriate culture conditions. Moreover, the study by Grabert et al. has indicated that CD115 (receptor for M-CSF, also known as cFms and CSFR1) may be used as a potential universal marker for cells of the mononuclear phagocyte system (22).

Many factors, including cytokines, growth factors and local conditions, are able to modulate differentiation and activity of myeloid lineage cells (osteoclasts, macrophages and dendritic cells) at least in part by acting on the polarization of the common trilineage progenitor (also named MODP) (4, 23, 24). Moreover, these three lineages comprise a variety of their functional subsets with either protective or harmful effects during immune responses (4, 9, 10). As a result, skewing towards a certain differentiation pathway may play an important role in the pathophysiology of inflammatory, infectious or autoimmune disorders. Among other signaling pathways, Notch signaling is an important, yet incompletely understood, pathway for which multiple roles in lineage commitment of myeloid cells were proposed (25). In mammals, the Notch signaling pathway comprises receptors Notch 1 through 4, and ligands Jagged (JAG) 1, JAG 2, Delta-like (DLL) 1, DLL 3 and DLL 4. Notch receptors and ligands are transmembrane proteins used for neighboring cell communication, which upon interaction result in the cleavage of the Notch intracellular domain (NICD) of the respective receptor by the γ-secretase complex (26, 27). Signaling continues by translocation of the NICD to the nucleus, displacement of transcriptional repressors and association of the NICD with other transcriptional factors, including recombination signal binding protein for immunoglobulin kappa J region (RBP-J). The resulting complex then mediates the transcription of Notch canonical target genes which include members from the HES and HEY families, as well as a number of lineage-specific genes involved in lineage fate determination and function (28). The result of signaling through Notch depends on many factors which include timing, location and the type of ligand and receptor interacting. Furthermore, the same ligand may elicit different effects depending whether it is coming from the same cell (cis signaling) or from a different cell (trans signaling) (29). The role of Notch signaling is well established as essential for T cell and marginal zone B cell lineage commitment, regulation of mature T cell responses, and its role in other hematopoietic cells is coming into view (30–39). However, through conflicting findings, there is still a lot to learn about its role in the myeloid lineage (25).

In our study, we induced differentiation of osteoclasts, macrophages and dendritic cells from the common trilineage progenitor isolated from the bone marrow of conditional inducible transgenic mice. Notch 1 signal activation (NICD1 overexpression) or Notch signaling inhibition (RBP-J deletion) in the trilineage progenitor was induced *in vitro* by addition of 4-hydroxytamoxifen (4-OHT) resulting in CX3CR1-driven Cre-mediated recombination. Our experimental setting allowed us to define a cell autonomous response to Notch signaling at the stage of the trilineage progenitor. Under basal and inflammatory conditions, we studied the effect of Notch modulation on differentiation and activation marker expression of the three lineages as well as on the function of osteoclasts, macrophages and dendritic cells in the context of bone resorption, phagocytosis and antigen presentation, respectively. NICD1 overexpression decreased osteoclast differentiation gene expression and reduced the number of functional tartrate-resistant acid phosphatase (TRAP)^+^ bone resorbing cells. Activation of Notch 1 pathway also reduced the functional properties of dendritic cells to acquire an activation phenotype and present antigens. On the other hand, disruption of the Notch canonical pathway resulted in a higher number and size of osteoclasts, with upregulation of *Ctsk* gene expression and RANK protein expression. RBP-J deletion in macrophages did not affect cell proliferation, but highly stimulated *Il12b* gene expression. Moreover, Notch signaling inhibition generally stimulated TRAP activity by osteoclasts and phagocytosis by macrophages, especially under inflammatory conditions.

## Materials and Methods

### Mice

All animal experiments in this study were conducted under protocols approved by the national Ethics Committee (EP 182/2018). Mice were maintained at the animal facility of the Croatian Institute for Brain Research, University of Zagreb School of Medicine (Zagreb, Croatia) under standard housing conditions. Male mice (2.5 to 3.5 months old) were used to harvest bone marrow cells in all experiments. For characterization of the common trilineage progenitor, and experiments with ligand stimulation and Notch neutralization we used wild type C57Bl/6 mice. To generate mice overexpressing Notch 1 signaling in the progenitor cell we used CX3CR1CreERT2 mice (B6.129P2(C)-*Cx3cr1^tm2.1(cre/ERT2)Jung^*/J from The Jackson Laboratory, stock no. 020940) and bred them with NICD1 floxed mice (*Gt(ROSA)26Sor^tm1(Notch1)Dam^*/J from The Jackson Laboratory, stock no. 008159) to produce CX3CR1CreERT2/NICD1. The offspring (heterozygous for both transgenes) were bred again with NICD1 floxed mice to obtain Cre^+^ and Cre^−^ littermates, the latter used as controls. To generate mice in which Notch signaling is silenced in the progenitor cell we used CX3CR1CreERT2 mice and bred them with RBP-J floxed mice (Rbpj^tm1Hon^, provided by professor Tasuku Honjo, Kyoto University) to produce CX3CR1CreERT2/RBP-J mice. The offspring were bred again with RBP-J floxed mice to obtain Cre^+^ and Cre^−^ littermates, homozygous for the floxed RBP-J allele. For visualizing tamoxifen-inducible Cre-mediated recombination, we crossed CX3CR1CreERT2 mice with the Ai9 reporter mice (B6.Cg-*Gt(ROSA)26Sor^tm9(CAG-tdTomato)Hze^*/J from The Jackson laboratory, stock no. 007909) to produce CX3CR1CreERT2/Ai9 mice. We determined the genotype for CX3CR1CreERT2, NICD1, RBP-J and Ai9 by PCR. For antigen presentation assay we used CD8^+^ T-cells isolated from spleens of OT-1 mice (C57BL/6-Tg(TcraTcrb)1100Mjb/J from The Jackson laboratory, stock no. 003831).

### Fluorescence-Activated Cell Sorting

Single cell suspensions were prepared by flushing femurs and tibias with staining medium [phosphate-buffered solution containing 2% fetal bovine serum (PBS/2% FBS)], followed by red blood cell lysing. Cells were labeled with commercially available monoclonal antibodies for phenotyping and cell sorting, incubated for 30 minutes at 4°C in the dark and washed with staining medium. For labeling we used a mixture of antibodies specific for lymphoid markers (anti-B220 FITC, clone RA3-6B2; anti-CD3e FITC, clone 145-2C11; anti-NK1.1 FITC, clone PK136), myeloid markers (anti-CD11b APC/Fire™750, clone M1/70; anti-Ly6G PerCP-eFluor710, clone 1A8; anti-CD115 PE/Cyanine7, clone AFS98), the panleukocyte marker (anti-CD45 APC, clone 30-F11), chemokine receptor (anti-CX3CR1 BV421, clone SA011F11), Notch receptors (anti-Notch1 APC, clone HMN1-12; anti-Notch2 PE, clone 16F11, anti-Notch3 PE, clone HMN3-133; anti-Notch4 APC, clone HMN4-14) from BioLegend (San Diego, CA, USA), eBiosciences (San Diego, CA, USA), R&D Systems (Bio-Techne, Abingdon, UK) or BD Biosciences (San Jose, CA, USA). We used 4’,6-diamidino-2-phenylindole (DAPI, Sigma) staining to exclude dead cells. Gates were set according to unstained, fluorescence minus one and/or isotype controls. Stained cells were analyzed on BD FACSAria II (BD Biosciences) instrument, the progenitor population (CD45^+^Ly6G^−^CD3^−^B220^−^NK1.1^−^CD11b^–/lo^CD115^+^) was sorted using gating strategies previously described (2, 16) and collected in tubes containing α-minimum essential medium (α-MEM)/10% FBS (Gibco, Thermo Fisher Scientific). For antigen presentation assay, we isolated CD8^+^ T cells from spleens of OT-1 mice. Single cell suspensions were prepared from harvested spleens and frozen in liquid nitrogen, for later use, in α-MEM/30% FBS and 10% dimethyl sulfoxide (DMSO, Sigma-Aldrich). After thawing, the cells were resuspended in 10× volume of α-MEM/10% FBS, plated and incubated for 2 hours at 37°C. Non-adherent cells were then collected and labeled with monoclonal antibodies as described above. For labeling, we used a mixture of antibodies specific to lymphoid markers (anti-CD3e APC-eFluor780, clone 145-2C11; anti-CD8 APC, clone 53-6.7) and the panleukocyte marker (anti-CD45 BV510, clone 30-F11). Sorting of CD45^+^CD3^+^CD8^+^ cells was performed on BD FACSAria II and cells were collected in RPMI/10% FBS. Sorting purity, verified by reanalyzing sorted cells, was higher than 99% for all experiments. The data were analyzed using the FlowJo software (TreeStar, Ashland, OR, USA).

### Cell Culture and Differentiation

Sorted progenitors were plated into 96-, 48- or 24-well plates at a density of 5×10^3^ – 2×10^4^ cells/well, 2.5×10^3^ – 2×10^4^ cells/well and 7.5×10^3^ – 4×10^4^ cells/well, for osteoclast, macrophage and dendritic cell differentiation, respectively, depending on the experiment. For osteoclast differentiation, cells were cultured in α-MEM/10% FBS supplemented with 30 ng/mL recombinant mouse (rm) macrophage colony-stimulating factor (M-CSF; R&D Systems, NE Minneapolis, MN, USA) and 30 ng/mL rm receptor activator of nuclear factor κB ligand (RANKL; R&D Systems); for macrophage differentiation, cells were cultured in α-MEM/10% FBS supplemented with 30 ng/mL rmM-CSF; for dendritic cell differentiation, cells were cultured in RPMI/10% FBS supplemented with 20 ng/mL rm interleukin 4 (IL-4; R&D Systems) and 20 ng/mL rm granulocyte-macrophage colony-stimulating factor (GM-CSF; R&D Systems). All wells were supplemented with 1 µM 4-OHT (Sigma-Aldrich) to induce Cre-mediated recombination. For some experiments, supplementation with 100 ng/mL of lipopolysaccharides (LPS) from *Escherichia (E.) coli* O111:B4 (Sigma-Aldrich) was used.

At the culture endpoint (3 days for dendritic cells, 4 days for macrophages and osteoclasts), the cells were fixed with 4% paraformaldehyde in PBS and stained for TRAP expression (Leukocyte acid phosphatase kit; Sigma-Aldrich) or hematoxylin according to the manufacturer’s instructions. The wells were scanned using Axiovert 200 light microscope (Carl Zeiss Microscopy, Jena, Germany) at 100× magnification connected to CCD camera and ZEISS ZEN 3.3 lite software (Panorama imaging). The cells were counted using CellProfiler 3.0 software (40) set to count objects of diameter greater than 6 or 8.5 µm for macrophages and dendritic cells, respectively. TRAP^+^ multinucleated cells with more than three nuclei with a diameter larger than 37 µm were counted as osteoclasts while those larger than 125 µm were counted as large osteoclasts.

### Modulation of Notch Signaling Using Immobilized Ligands and Neutralizing Antibodies

To stimulate the Notch signaling pathway in the differentiating trilineage progenitor, we used immobilized JAG 1:Fc fusion protein, as previously described (41). We added 50 μL per well (96-well plate) of anti-human IgG Fc antibody (BioLegend) in PBS at a concentration of 10 μg/mL. The antibody was incubated at 4°C overnight. The wells were washed 3× with PBS, then 50 μL of recombinant human JAG 1:Fc (R&D Systems) was added at a concentration of 10 μg/mL and incubated for 2 hours at 37°C. The wells were washed again 3× with PBS and PBS was kept in the wells until seeding to prevent drying. For Notch neutralization, we used anti-Notch 1 neutralizing antibodies (AF1057, R&D Systems), targeting the negative regulatory region of the Notch 1 receptor, preventing cleavage and signal transmission. Antibodies were added to the cell culture medium at a concentration of 20 μg/mL. Cells isolated from the bone marrow of C57Bl/6 mice were used as the source of the trilineage progenitor and were seeded and differentiated as described above.

### Phenotyping

For phenotyping experiments, cells were cultured in osteoclast, macrophage and dendritic cell polarizing conditions as described above for 48 hours with or without the addition of 100 ng/mL of LPS 12 hours prior to harvesting. For cell harvesting, TrypLE Express enzyme (Gibco, Thermo Fisher Scientific) was added to the wells and incubated for 15 min at 37°C. Cell pellets were then labeled with antibodies and analyzed by flow cytometry as described above. For labeling we used a mixture of antibodies to the panleukocyte marker (anti-CD45 BV510, clone 30-F11), myeloid markers (anti-CD11b FITC, clone M1/70; anti-CD11c APC, clone N418; anti-CD115 biotinylated or PE/Cyanine7, clone AFS98; anti-F4/80 APC-eFluor780, clone BM8; anti-CD64 (FcRγ) PerCP/Cyanine5.5, clone X54-5/7.1), MHC and co-stimulatory markers (anti-MHCII PE, clone; anti-CD86 PE/Cyanine7, clone GL-1), osteoclastogenic marker (anti-CD265 (RANK) PE, clone R12-31), and streptavidin coupled to PE-CF594. We used DAPI staining to exclude dead cells. Gates were set according to unstained, fluorescence minus one and/or isotype controls. The cells were acquired on BD FACSAria II and the data analyzed using the FlowJo software.

### Quantitative PCR Gene Expression Analysis

For quantitative PCR (qPCR), total RNA was extracted from cells cultured for 3 days using Trizol reagent (Applied Biosystems, Thermo Fisher Scientific) according to the manufacturer instructions. cDNA was then reverse transcribed using High-Capacity RNA-to-cDNA Kit (Applied Biosystems). The amount of cDNA corresponding to 20 ng of reversely transcribed RNA was amplified in triplicates by ABI Prism 7500 system (Applied Biosystems), using TaqMan Gene Expression Master mix and commercially available TaqMan Gene Expression Assays (Applied Biosystems) for mouse osteoclast differentiation genes *Fos* (Assay ID: Mm00487425_m1) and *Ctsk* (Assay ID: Mm00484039_m1); macrophage differentiation genes *Cd68* (Assay ID: Mm03047343_m1) and *Il12b* (Assay ID: Mm99999067_m1); dendritic cell differentiation genes *Cd80* (Assay ID: Mm00711660_m1) and *Cd86* (Assay ID: Mm00444543_m1); Notch target genes *Hes1* (Assay ID: Mm01342805_m1), *Hey1* (Assay ID: Mm00468865_m1) and *Bcl2* (Assay ID: Mm00477631_m1); and housekeeping gene *Hmbs* (Assay ID: Mm01143545_m1). Gene expression was calculated from the relative standard curve of gene expression in the calibrator sample (cDNA from Cre^−^ cultures) and normalized to the expression level of the housekeeping gene *Hmbs* (hydroxymethylbilane synthase). As previously described, the methodological studies of qPCR analysis suggest that the minimal difference in gene expression that is statistically significant and approximately 2-fold different compared with control, could be considered as biologically significant (42).

### Bone Resorption Assay

Osteoclasts were tested for their bone resorbing ability. Sorted progenitor cells were plated on square shaped bovine cortical bone slices 4.4×4.4×0.2 mm (10^4^ cells/slice) in osteoclastogenic culture as described above. After 14 days of culturing, slices were fixed and stained for TRAP expression. Then they were sonicated in 0.25 M NH_4_OH for 5-10 minutes and stained with 1% toluidine blue in 1% borax buffer for 1 minute, to visualize resorption pits using the Axiovert 200 light microscope.

### Phagocytosis Assay

Macrophages were tested for their ability to phagocytose pHrodo *E. coli* bacterial particles (Invitrogen) containing rhodamine, which is colorless at neutral pH and activated at the low pH inside phagocytic vesicles. Cells were cultured in 24-well plates in α-MEM/10% FBS supplemented with 30 ng/mL rmM-CSF for a total of 4 days. 12 hours before harvesting 100 ng/mL of LPS or PBS was added to the wells. Cells were harvested using TrypLE Express enzyme as described above, counted and plated at 10^4^ cells/well in 100 µL α-MEM/10% FBS in a 96-well plate and incubated at 37°C for 2 hours for the cells to adhere. 10 µg of pHrodo *E. coli* particles were added to the wells and incubated at 37°C for 2 hours. Phagocytosed particles were visualized by fluorescent microscopy (Axiovert 200 light microscope with Colibri 7 as the light source, Carl Zeiss Microscopy, Jena, Germany). The cells were counted using CellProfiler 3.0 software set to count fluorescent cells of diameter larger than 8.5 µm.

### Antigen Presentation Assay

Dendritic cells were tested for their ability to process and present antigen to T cells, leading to their activation and proliferation. The cells were cultured in 48-well plates in RPMI/10% FBS supplemented with 20 ng/mL rmIL-4 and 20 ng/mL rmGM-CSF for 4 days. 12 hours before harvesting 100 ng/mL of LPS was added to the wells. The cells were incubated with 100 ng/mL SIINFEKL (Sigma-Aldrich) for 2 hours at 37°C. Dendritic cells were then harvested by collecting the cell supernatant and lightly pipetting up and down, washed and plated in 100 µL of RPMI/10% FBS in 96-well U-bottom plates at 2.5×10^4^ cells/well. FACS sorted CD8^+^ T cells from OT-1 mice were stained with 5-(and-6)-carboxyfluorescein diacetate succinimidyl ester (CFSE, Thermo Fisher Scientific). The cells were incubated with 5 μM CFSE in PBS/5% FBS for 5 min at room temperature, washed in PBS/5% FBS and counted. A total of 4×10^4^ cells were added to each well containing dendritic cells. After 48 hours, cells were harvested, and T cell proliferation was analyzed by flow cytometry (BD FACSAria II) by analyzing CFSE fluorescence in daughter cells since its concentration halves with every cell division. Unstimulated (undivided) CFSE-labeled T cells were used as a reference.

### Statistical Analysis

The results were statistically analyzed using MedCalc Statistical Software version 13.1.2 (MedCalc Software, Ostend, Belgium). Kolmogorov–Smirnov test was used to verify normality of data distribution. Results are presented as individual values (circles) and plotted as box-and-whisker diagrams, where middle horizontal lines represent medians, boxes represent the interquartile range (IQR), and whiskers represent 1.5 times the IQR. For some measurements (indicated in figure legends), values were normalized to the average of the control group. qPCR gene expression was assessed in technical replicates of the representative experiment. Differences between groups were analyzed by Mann–Whitney U-test or by the non-parametric Kruskal–Wallis test followed by Conover test for group-to-group comparisons. In all experiments α-level was set at 0.05.

## Results

### Trilineage Progenitor for Osteoclasts, Macrophages and Dendritic Cells

**Figure 1:**
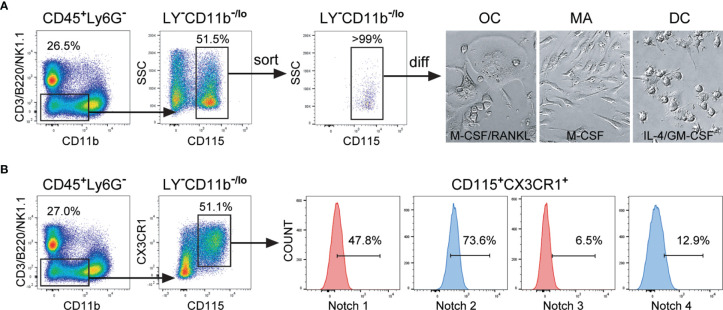
Expression of Notch receptors by the common trilineage progenitor isolated from the bone marrow cells of C57BL/6 mice. Bone marrow cells were flushed from hind limb long bones and the common trilineage progenitors were identified as CD45^+^Ly6G^−^CD3^−^B220^−^NK1.1^−^CD11b^–/lo^CD115^+^ cells using flow-cytometry. **(A)** Trilineage progenitor cells have the ability to differentiate into osteoclasts (OC), macrophages (MA) and dendritic cells (DC), under appropriate culture conditions. Sorting efficiency of the common trilineage progenitor was above 99%. Differentiation of OCs was induced by receptor activator of nuclear factor κB ligand (RANKL) and macrophage colony-stimulating factor (M-CSF); MAs by M-CSF; DCs by interleukin 4 (IL-4) and granulocyte-macrophage colony-stimulating factor (GM-CSF). Representative bright field images show typical morphology of differentiated unstained OCs, MAs and DCs. **(B)** Trilineage progenitor cells express the chemokine receptor CX3CR1, justifying the use of CX3CR1CreERT2 mice to modulate Notch signaling. In addition, the progenitor population expresses different percentages of Notch receptors (Notch 1 through Notch 4), as determined by flow cytometry. Representative histograms for Notch receptor expression on CX3CR1^+^ trilineage progenitors are shown. LY^−^ – CD3^−^B220^−^NK1.1^−^.

In line with other authors and our previous work (2, 13, 18), we identified the common trilineage progenitor (also named MODP) as CD45^+^Ly6G^−^CD3^−^B220^−^NK1.1^−^CD11b^–/lo^CD115^+^ subset, which comprises around 5% of total hematopoietic CD45^+^ cells in the mouse bone marrow. The applied gating strategy included delineation of live bone marrow cells, then comprised non-granulocyte (Ly6G^−^) hematopoietic (CD45^+^) cells, followed by gating of non-lymphoid (CD3^−^B220^−^NK1.1^−^) cells with low expression of CD11b (CD11b^−/lo^) (**Figure 1A** and **Supplementary Figure 1**). Progenitors expressing the M-CSF receptor (CD115^+^) were sorted at >99% purity and plated in cell culture to show their trilineage potency (**Figure 1A**).

Upon *in vitro* differentiation, the cells showed the typical morphology of the respective lineage (**Figure 1A**). Mature osteoclasts appeared as adherent, mostly multinucleated cells with abundant cytoplasm. In contrast, macrophages were mostly elongated, firmly adherent mononuclear cells. Dendritic cells became progressively less adherent with maturation and finally appeared as loosely attached round shaped cells with fine processes. The majority of CD45^+^Ly6G^−^CD3^−^B220^−^NK1.1^−^CD11b^–/lo^CD115^+^ cells express the chemokine receptor CX3CR1, justifying the use of CX3CR1CreERT2 mice to modulate Notch signaling in trilineage progenitor commitment and differentiation (**Figure 1B**). These CX3CR1^+^ trilineage progenitors express different amounts of analyzed Notch receptors, amongst which Notch 1 and Notch 2 are the most abundantly expressed, at about 50% and 75% respectively (**Figure 1B**).

We confirmed that the trilineage progenitor is susceptible to signaling through Notch by stimulating Notch receptors with the immobilized JAG 1 ligand. Seeding isolated trilineage progenitors on JAG 1 coated plates resulted in inhibition of differentiation in all three lineages (**Supplementary Figure 2**). This effect was reversed by blocking signaling through Notch 1 with anti-Notch 1 neutralizing antibodies, most obvious in osteoclastogenic cultures. The finding suggests that the ligand JAG 1, abundant in the bone marrow milieu (29), conveys an inhibitory effect on the trilineage progenitor, by interacting with the Notch 1 receptor.

### Differentiation From the Trilineage Progenitor Under Notch Signaling Modulation

**Figure 2:**
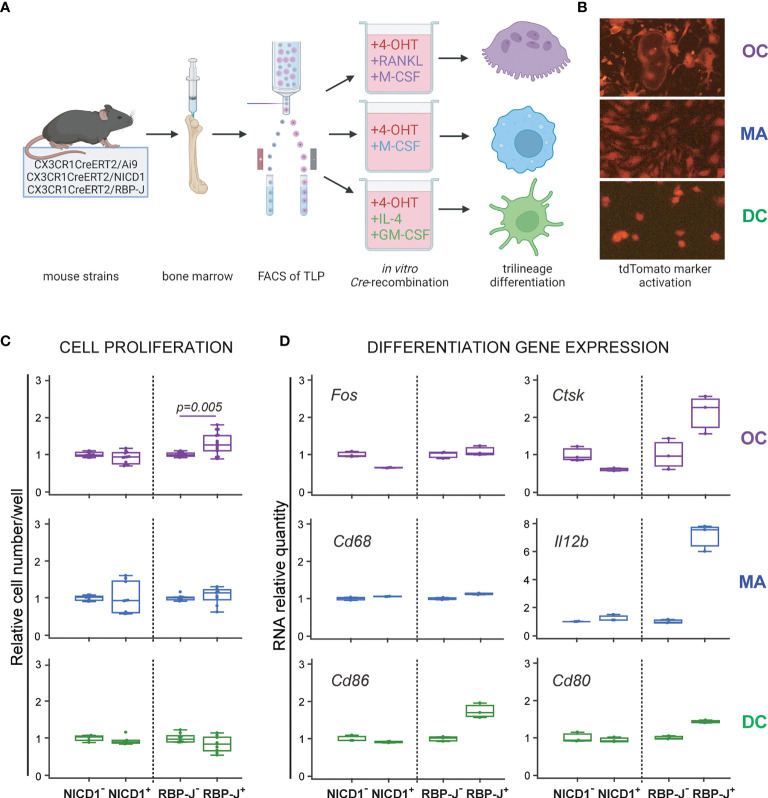
Differentiation potential of the common trilineage progenitor (TLP) isolated from bone marrow cells of CX3CR1CreERT2/Ai9 (Ai9), CX3CR1CreERT2/NICD1 (NICD1) and CX3CR1CreERT2/RBP-J (RBP-J) mice. **(A)** Experimental design of trilineage differentiation. Bone marrow cells were flushed from hind limb long bones and the common TLPs were identified as CD45^+^Ly6G^−^CD3^−^B220^−^NK1.1^−^CD11b^–/lo^CD115^+^ cells using flow-cytometry. TLPs were sorted and plated under appropriate culture conditions. Differentiation of osteoclasts (OC) was induced by receptor activator of nuclear factor κB ligand (RANKL) and macrophage colony-stimulating factor (M-CSF); macrophages (MA) by M-CSF; dendritic cells (DC) by interleukin 4 (IL-4) and granulocyte-macrophage colony-stimulating factor (GM-CSF). Cre-mediated recombination in *Cre*^+^ (NICD1^+^ or RBP-J^+^) littermates was induced by the addition of 4-hydroxytamoxifen (4-OHT). *Cre*^−^ (NICD1^−^ or RBP-J^−^) littermates treated with 4-OHT were used as controls. Created with BioRender.com **(B)** Visualization of Cre-mediated recombination by using TLP from CX3CR1CreERT2/Ai9 mice. Representative fluorescent images show tdTomato^+^ differentiated cells with typical morphology of OCs, MAs and DCs. **(C)** Quantification of cells differentiated from NICD1 and RBP-J mice. Cells were differentiated from *Cre*^+^ and *Cre*^−^ littermates, fixed at culture day 3-4, stained and quantified using CellProfiler. Cell numbers were normalized to the appropriate *Cre*^−^ control in each experiment; pooled data from three independent experiments are shown (n=9-12). Statistically significant difference was determined at p<0.05 between corresponding *Cre*^+^ and *Cre*^−^ groups, Mann–Whitney U-test. **(D)** qPCR analysis of differentiation gene expression for OCs (*Fos*, *Ctsk*), MAs (*Cd68*, *Il12b*) and DCs (*Cd86*, *Cd80*). Cells were harvested at culture day 3 for RNA isolation. Gene expression was normalized using *Hmbs* housekeeping gene and presented as relative gene expression normalized to the appropriate *Cre*^−^ control; technical replicates for the representative experiment are shown (n=3). Expression difference of approximately 2-fold is considered biologically significant. **(C**, **D)** Individual values are presented; horizontal lines represent the median, boxes represent the interquartile range (IQR), whiskers represent 1.5 times the IQR.

To modulate Notch signaling during trilineage differentiation, we used conditional and inducible transgenic CX3CR1CreERT2/NICD1 (NICD1^+^) and CX3CR1CreERT2/RBP-J (RBP-J^+^) mice as the source of the progenitor population. The tamoxifen-inducible Cre recombinase is under the control of CX3CR1 gene promoter and is expressed in cells of the monocyte/macrophage lineage, including the trilineage progenitor (**Figure 1B**). When CreERT2 mice are bred with mice containing *loxP*-flanked (floxed) sequences, Cre-mediated recombination results in floxed sequence deletion. Therefore Cre-mediated recombination in NICD1^+^ mice results in STOP codon deletion and NICD1 overexpression, mimicking constitutively active Notch 1 receptor. In RBP-J^+^ mice, on the other hand, Cre-mediated recombination leads to RBP-J sequence deletion and disruption of downstream canonical Notch signaling. To induce Cre-mediated Notch 1 signal activation or Notch signaling disruption, 4-OHT was added to cultured sorted trilineage progenitors, in addition to appropriate differentiation factors (**Figure 2A**). The Ai9 reporter mouse line expresses red fluorescent tdTomato protein in cells that express active Cre recombinase and their progeny. By using CX3CR1CreERT2Ai9 reporter mice, we showed effective Cre-mediated recombination following *in vitro* 4-OHT administration in differentiation of osteoclasts, macrophages and dendritic cells (**Figure 2B**). Cells from *Cre* negative littermates (NICD1^−^, RBP-J^−^ and Ai9^−^) treated with 4-OHT were used as controls. We confirmed effective Cre-mediated recombination by analyzing Notch transcriptional target genes in differentiating cells. Stimulation of Notch 1 pathway resulted in upregulation of *Hes1*, *Hey1* and *Bcl2* with the greatest magnitude of change observed for *Hes1* expression in osteoclasts and macrophages, increasing several hundredfold (**Supplementary Figure 3**). Downregulation of the target genes was detected with canonical pathway (RBP-J) deletion, with the exception of *Hes1* expression in RBP-J^+^ macrophages which was upregulated compared to the respective RBP-J^−^ control. However, the degree of upregulation was significantly (25-fold) lower than in NICD1^+^ macrophages. As *Hes1* can be under its own negative-feedback regulation, and is downstream to other signaling pathways apart from Notch (29), this finding could suggest that RBP-J deletion releases other critical pathways in macrophage differentiation.

Effects of Notch modulation were assessed by plating the trilineage progenitor cells isolated from the two transgenic strains (NICD1^+^ and RBP-J^+^) and their respective controls in osteoclast, macrophage and dendritic cell polarizing conditions (**Figure 2A**). Proliferation was assessed by the number of differentiated cells at the culture endpoint and differentiation was assessed by expression of lineage-specific differentiation genes at culture day 3. Notch signaling inhibition enhanced osteoclastogenesis, resulting in a higher number of cells per well, whereas Notch 1 signaling stimulation showed no significant effect on the total cell number (**Figure 2C**). Moreover, Notch deletion increased relative gene expression of *Ctsk* in osteoclast progenitors, whereas NICD1 overexpression conversely reduced *Ctsk* gene expression (**Figure 2D**). Notch modulation did not seem to affect the number of differentiated macrophages or dendritic cells (**Figure 2C**). However, Notch deletion resulted in the upregulation of *Il12b* expression in macrophages as well as *Cd86* expression in dendritic cells (**Figure 2D**). Overall, the results indicate that canonical Notch signaling may have an inhibitory role in the trilineage progenitor differentiation, especially in the osteoclast lineage.

### Effect of Notch Modulation on Osteoclast, Macrophage and Dendritic Cell Immunophenotype Under LPS Stimulation

**Figure 3:**
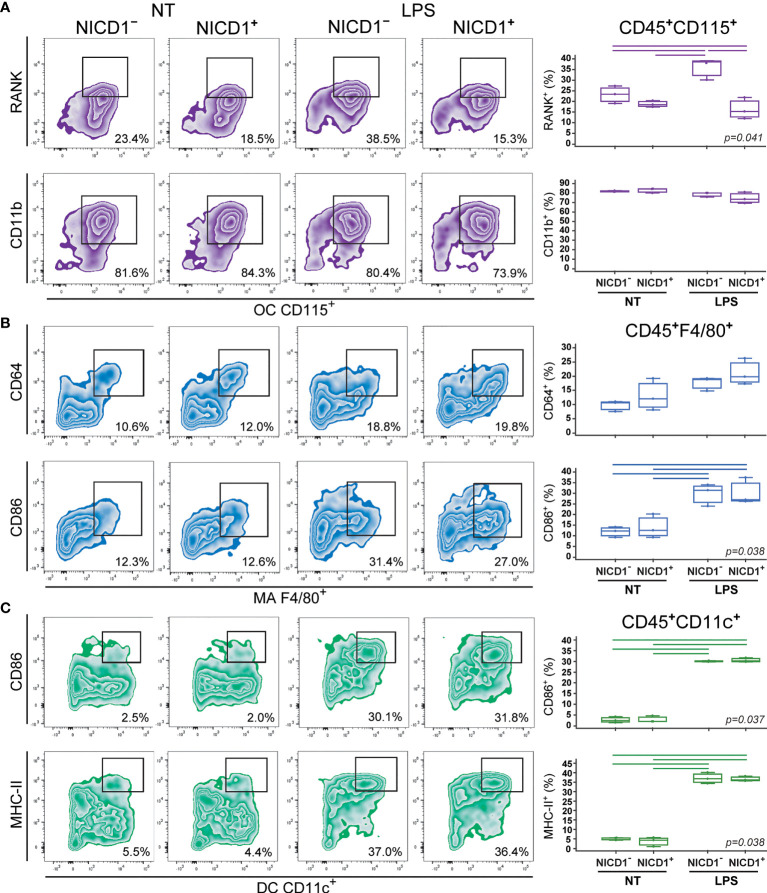
Phenotype of osteoclasts (OCs), macrophages (MAs) and dendritic cells (DCs) differentiated from the common trilineage progenitor (TLP) of CX3CR1CreERT2/NICD1 mice. Bone marrow cells were flushed from hind limb long bones and the common TLPs were identified as CD45^+^Ly6G^−^CD3^−^B220^−^NK1.1^−^CD11b^–/lo^CD115^+^ cells using flow-cytometry. TLPs were sorted and plated under appropriate culture conditions. Differentiation of OCs was induced by receptor activator of nuclear factor κB ligand (RANKL) and macrophage colony-stimulating factor (M-CSF); MAs by M-CSF; DCs by interleukin 4 (IL-4) and granulocyte-macrophage colony-stimulating factor (GM-CSF). Cre-mediated recombination in *Cre*^+^ (NICD1^+^) littermates was induced by the addition of 4-hydroxytamoxifen (4-OHT). *Cre*^−^ (NICD1^−^) littermates treated with 4-OHT were used as controls. Lipopolysaccharides from *E.coli* (LPS) were added 12h before harvesting, to induce the inflammatory response; non-treated (NT) cells were used for comparison. Cells were harvested at culture day 2. **(A)** Expression of RANK and CD11b on CD115^+^ osteoclast-committed progenitors stimulated by LPS. Representative dot plots showing RANK^+^CD115^+^ and CD11b^+^CD115^+^ subpopulations. **(B)** Expression of CD64 and CD86 on F4/80^+^ macrophage-committed progenitors stimulated by LPS. Representative dot plots showing CD64^+^F4/80^+^ and CD86^+^F4/80^+^ subpopulations. **(C)** Expression of CD86 and MHC-II on CD11c^+^ dendritic cell-committed progenitors stimulated by LPS. Representative dot plots showing CD86^+^CD11c^+^ and MHC-II^+^CD11c^+^ subpopulations. **(A–C**, *right***)** Individual values of biological triplicates are presented; horizontal lines represent the median, boxes represent the interquartile range (IQR), whiskers represent 1.5 times the IQR. Statistically significant difference was determined at p<0.05, Kruskal–Wallis test followed by Conover test for group-to-group comparisons (lines denote significant difference between groups).

**Figure 4:**
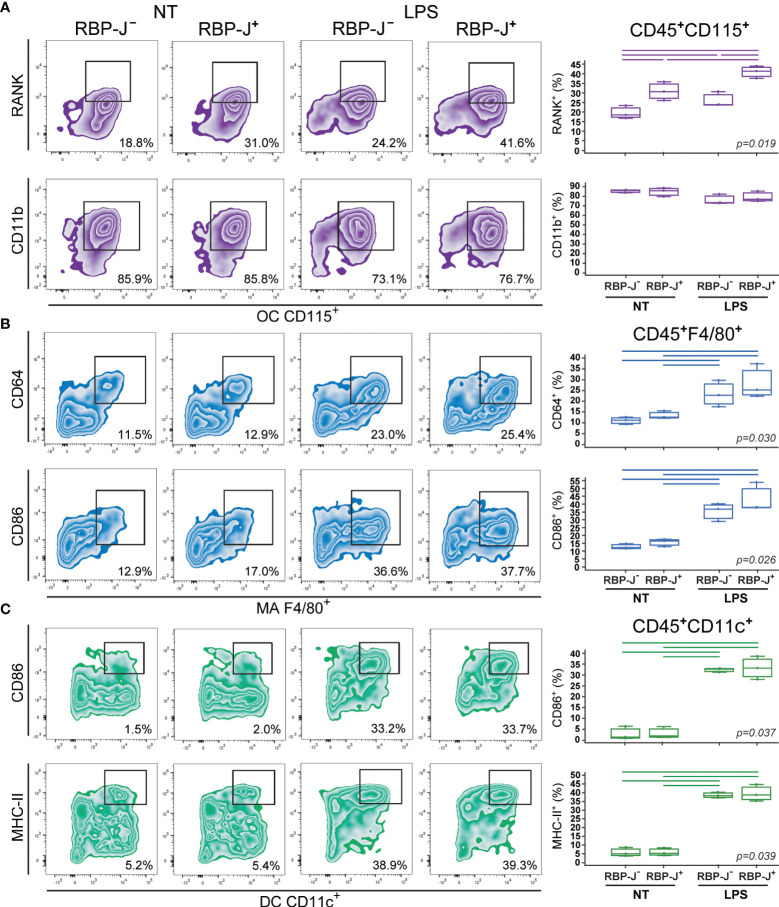
Phenotype of osteoclasts (OCs), macrophages (MAs) and dendritic cells (DCs) differentiated from the common trilineage progenitor (TLP) of CX3CR1CreERT2/RBP-J mice. Bone marrow cells were flushed from hind limb long bones and the common TLPs were identified as CD45^+^Ly6G^−^CD3^−^B220^−^NK1.1^−^CD11b^–/lo^CD115^+^ cells using flow-cytometry. TLPs were sorted and plated under appropriate culture conditions. Differentiation of OCs was induced by receptor activator of nuclear factor κB ligand (RANKL) and macrophage colony-stimulating factor (M-CSF); MAs by M-CSF; DCs by interleukin 4 (IL-4) and granulocyte-macrophage colony-stimulating factor (GM-CSF). Cre-mediated recombination in *Cre*^+^ (RBP-J^+^) littermates was induced by the addition of 4-hydroxytamoxifen (4-OHT). *Cre*^−^ (RBP-J^−^) littermates treated with 4-OHT were used as controls. Lipopolysaccharides from *E.coli* (LPS) were added 12h before harvesting, to induce the inflammatory response; non-treated (NT) cells were used for comparison. Cells were harvested at culture day 2. **(A)** Expression of RANK and CD11b on CD115^+^ osteoclast-committed progenitors stimulated by LPS. Representative dot plots showing RANK^+^CD115^+^ and CD11b^+^CD115^+^ subpopulations. **(B)** Expression of CD64 and CD86 on F4/80^+^ macrophage-committed progenitors stimulated by LPS. Representative dot plots showing CD64^+^F4/80^+^ and CD86^+^F4/80^+^ subpopulations. **(C)** Expression of CD86 and MHCII on CD11c^+^ dendritic cell-committed progenitors stimulated by LPS. Representative dot plots showing CD86^+^CD11c^+^ and MHCII^+^CD11c^+^ subpopulations. **(A–C**, *right*) Individual values of biological triplicates are presented; horizontal lines represent the median, boxes represent the interquartile range (IQR), whiskers represent 1.5 times the IQR. Statistically significant difference was determined at p<0.05, Kruskal–Wallis test followed by Conover test for group-to-group comparisons (lines denote significant difference between groups).

To determine further the role of Notch modulation, we assessed the activation phenotype of osteoclast-, macrophage- and dendritic cell-committed progenitors under inflammatory conditions. Differentiating cells were stimulated with LPS prior to analyzing their marker expression by flow cytometry at culture day 2. Each lineage was identified by the expression of a common marker CD115, F4/80 and CD11c for osteoclasts, macrophages, and dendritic cells, respectively (**Figures 3**, **4**). In addition, lineage specific activation markers were analyzed in the context of Notch modulation under the LPS treatment.

Osteoclast progenitors were identified by the expression of CD115 (cFms), receptor for M-CSF (16, 22). Expression of RANK, receptor activated by RANKL, on osteoclast progenitors showed the most evident and specific response to Notch modulation (**Figures 3A**, **4A**). NICD1 overexpression under LPS stimulation significantly downregulated the expression of RANK on CD115^+^ osteoclast progenitors, reducing the percentage of RANK^+^ cells by more than a half in Cre^+^ compared to Cre^–^ cultures (**Figure 3A**). Conversely, inhibition of Notch signaling significantly increased RANK expression, with additional stimulation by LPS (**Figure 4A**). Expression of the integrin CD11b was associated with *in vitro* osteoclast maturation and was shown to be stimulated by LPS (43, 44). However, modulation of Notch signaling did not seem to affect CD11b expression, irrespective of LPS treatment in our setting (**Figures 3A**, **4A**).

The signature marker of the mouse macrophage lineage, F4/80, was used to follow macrophage differentiation (**Figures 3B**, **4B**). In addition, CD64 (Fc-receptor) and CD86 (a costimulatory molecule) were analyzed on F4/80^+^ cells. Expression of CD64 is described to be upregulated upon inflammatory stimuli, such as LPS, and may be used as a marker of proinflammatory macrophages (45). In our experimental conditions, LPS indeed augmented the percent of CD64^+^ macrophages, but this response occurred irrespective of Notch modulation, in both NICD1 and RBP-J mice (**Figures 3B**, **4B**). Macrophage expression of CD86 is also induced by different cytokines and TLR ligands (7). As expected, CD86 was significantly induced by LPS, but without a clear association with Notch modulation (**Figures 3B**, **4B**). This may indicate that the Notch pathway is not the major regulator of macrophage activation by LPS.

Integrin CD11c is the most widely used defining marker for dendritic cells *in vivo* and *in vitro* (3, 9). Considering their major biological role in antigen presentation and T cell activation, dendritic cells were further characterized by the expression of the costimulatory molecule CD86 and the antigen-presenting molecule MHC-II. LPS treatment of dendritic cell-differentiating cultures significantly enhanced expression of CD86 and MHC-II in CD11c^+^ cells (**Figures 3C**, **4C**). This is in line with the known effect of LPS on dendritic cell maturation, marked by stronger presenting and activating functions. However, a similar magnitude of CD86 and MHC-II upregulation (approximately 10- and 6-fold, respectively) was observed in Cre^+^ and Cre^−^ cultures from both NICD1 and RBP-J transgenic strains (**Figures 3C**, **4C**). Again, it seems that the immunophenotype of maturing dendritic cells is not affected by *in vitro* Notch modulation.

### Increased Formation of Functional TRAP^+^ Osteoclasts Following Inhibition of Notch Signaling

**Figure 5:**
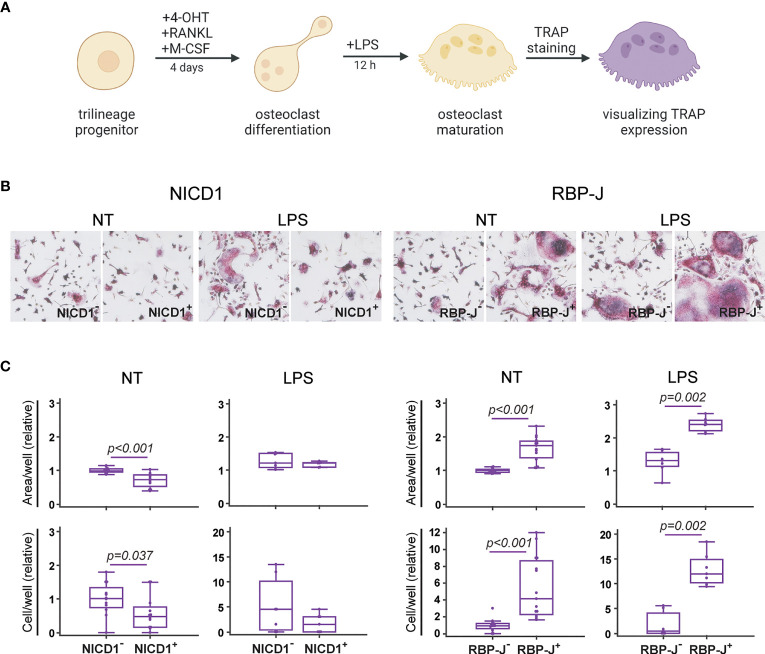
Functional characterization of osteoclasts differentiated from the common trilineage progenitor (TLP) isolated from bone marrow cells of CX3CR1CreERT2/NICD1 (NICD1) and CX3CR1CreERT2/RBP-J (RBP-J) mice. Bone marrow cells were flushed from hind limb long bones and the common TLPs were identified as CD45^+^Ly6G^−^CD3^−^B220^−^NK1.1^−^CD11b^–/lo^CD115^+^ cells using flow-cytometry. TLPs were sorted and plated under appropriate culture conditions. **(A)** Experimental design of osteoclast differentiation and functional testing. Osteoclast differentiation was induced by the addition of receptor activator of nuclear factor κB ligand (RANKL) and macrophage colony-stimulating factor (M-CSF). Cre-mediated recombination in *Cre*^+^ (NICD1^+^ or RBP-J^+^) littermates was induced by the addition of 4-hydroxytamoxifen (4-OHT). *Cre*^−^ (NICD1^−^ or RBP-J^−^) littermates treated with 4-OHT were used as controls. Lipopolysaccharides from *E.coli* (LPS) were added 12h before fixation to induce the inflammatory response. Expression of tartrate-resistant acid phosphatase (TRAP) in multinucleated cells was used as a functional assay. Created with BioRender.com **(B)** Representative images of culture wells with TRAP^+^ multinucleated osteoclasts differentiated from TLP under LPS stimulation. TRAP^+^ multinucleated cells with more than three nuclei and of diameter greater than 125 µm were counted as large osteoclasts (magnification 100×). **(C)** Quantification of multinucleated TRAP^+^ osteoclasts differentiated from TLP under LPS stimulation. Number and area of large multinucleated TRAP^+^ osteoclasts were normalized to the value of *Cre*^−^ controls without LPS stimulation. Pooled data from three independent experiments are shown (n=7-12). Individual values are presented; horizontal lines represent the median, boxes represent the interquartile range (IQR), whiskers represent 1.5 times the IQR. Statistically significant difference was determined at p<0.05, Mann–Whitney U-test.

As we previously showed (13), the common trilineage bone marrow progenitor, defined as the CD45^+^Ly6G^−^CD3^−^B220^−^NK1.1^−^CD11b^–/lo^CD115^+^ population, is able to differentiate into mature multinucleated osteoclasts under RANKL and M-CSF stimulation *in vitro* (**Figure 5A**). In addition to cell quantification and immunophenotyping, in this set of experiments we functionally characterized mature osteoclasts by their ability to resorb bone matrix and to express the TRAP enzyme under LPS treatment (**Figure 5A** and **Supplementary Figure 4**). The resorptive activity was tested on cortical bone slices, showing that the area of bone resorbing pits correlates with the area of TRAP^+^ osteoclasts (**Supplementary Figure 4**). We, therefore, used the number and area of large osteoclasts as a quantitatively more accurate measurement for *Cre*^+^ and *Cre*^−^ littermate comparison (**Figure 5B**).

Notch modulation showed a significant effect on the formation of bone resorbing osteoclasts, as seen by the resulting number of large multinucleated TRAP^+^ cells (**Figure 5C**). Specifically, Notch 1 overexpression resulted in generation of smaller osteoclasts, leading to a reduced total area covered with osteoclasts. Conversely, RBP-J deletion resulted in formation of a higher number of large TRAP^+^ osteoclasts (**Figure 5C**). Under LPS stimulation, known to enhance osteoclast differentiation *in vitro* when added to RANKL/M-CSF pretreated committed progenitors (46, 47), the effect of Notch modulation was still clearly visible. NICD1 signal activation suppressed, whereas Notch signal disruption enhanced formation of multinucleated TRAP^+^ osteoclasts under inflammatory conditions (**Figure 5C**). This functional confirmation, together with the effect on osteoclast-differentiation gene expression and on the percentage of RANK^+^CD115^+^ progenitors indicates that canonical Notch signaling acts as a negative regulator of osteoclast activity.

### Enhanced Macrophage Phagocytosis Following Inhibition of Notch Signaling

**Figure 6:**
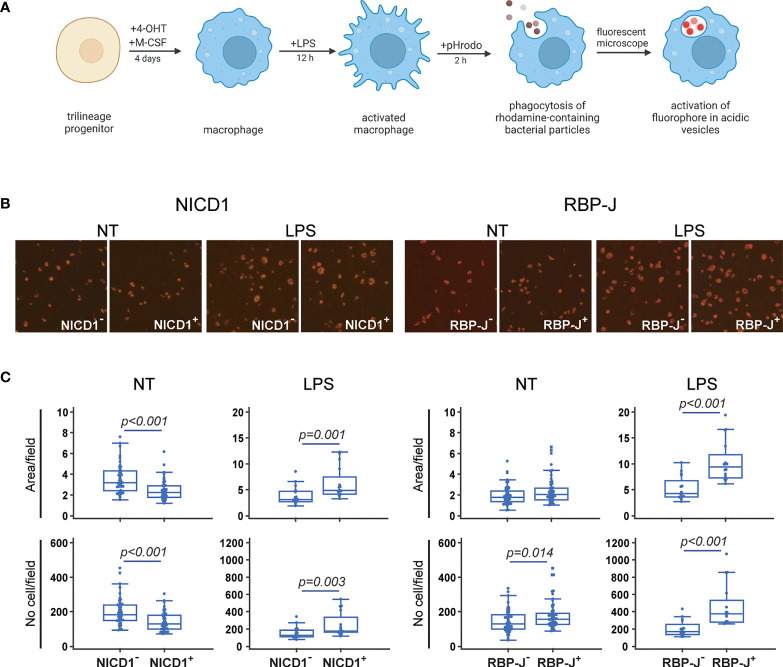
Functional characterization of macrophages differentiated from the common trilineage progenitor (TLP) isolated from bone marrow cells of CX3CR1CreERT2/NICD1 (NICD1) and CX3CR1CreERT2/RBP-J (RBP-J) mice. Bone marrow cells were flushed from hind limb long bones and the common TLPs were identified as CD45^+^Ly6G^−^CD3^−^B220^−^NK1.1^−^CD11b^–/lo^CD115^+^ cells using flow-cytometry. TLPs were sorted and plated under appropriate culture conditions. **(A)** Experimental design of macrophage differentiation and functional testing. Macrophage differentiation was induced by the addition of macrophage colony-stimulating factor (M-CSF). Cre-mediated recombination in *Cre*^+^ (NICD1^+^ or RBP-J^+^) littermates was induced by the addition of 4-hydroxytamoxifen (4-OHT). *Cre*^−^ (NICD1^−^ or RBP-J^−^) littermates treated with 4-OHT were used as controls. Lipopolysaccharides from *E.coli* (LPS) were added 12h before harvesting to induce the inflammatory response. Phagocytosis of rhodamine-containing bacterial particles was used as a functional assay. Created with BioRender.com **(B)** Representative images of culture wells with pHrodo^+^ macrophages differentiated from TLP under LPS stimulation. Activation of rhodamine in acidic vesicles after phagocytosis was detected as pHrodo^+^ macrophages (magnification 100×). **(C)** Quantification of pHrodo^+^ macrophages differentiated from TLP under LPS stimulation. Number and area of pHrodo^+^ macrophages were expressed per field of view. Pooled data from two independent experiments are shown (n=12-20). Individual values are presented; horizontal lines represent the median, boxes represent the interquartile range (IQR), whiskers represent 1.5 times the IQR. Statistically significant difference was determined at p<0.05, Mann–Whitney U-test.

To evaluate the functional ability of *in vitro* differentiated macrophages to perform phagocytosis, as the part of their major role in the immune response, we incubated mature cells with commercially available rhodamine-containing *E. coli* particles (**Figure 6A**). The particles are colorless in neutral pH, but when exposed to the acidic environment, like in the phagocytic vesicles, the fluorochrome is activated and emits fluorescence in the red spectrum. We assessed phagocytosis by visualizing the cells under the fluorescent microscope, and by counting the number and area covered with fluorescent particles in cultures generated from *Cre*^+^ and *Cre*^−^ littermates of CX3CR1CreERT2/NICD1 and CX3CR1CreERT2/RBP-J mice (**Figure 6B**).

By comparing macrophages isolated from different strains, we observed that Notch signaling inhibition increases the number and area of phagocytosed fluorescent particles, suggesting an enhanced phagocytic ability. In contrast, Notch 1 signal activation decreased the fluorescent cell number and area in NICD1^+^ compared to NICD1^–^ cultures (**Figure 6C**). LPS stimulation caused an apparent change in macrophage morphology, increasing individual cell-fluorescent area by approximately two fold (**Figure 6B**, quantification not shown). However, increased particle phagocytosis under LPS treatment, seen as a higher number and a larger area of fluorescence, was observed in both NICD1^+^ and RBP-J^+^ cultures compared to the respective controls (NICD1^–^ and RBP-J^–^ cultures) (**Figure 6C**). These ambiguous findings between LPS-untreated and LPS-treated cultures in NICD mice may indicate that the inhibitory effect of Notch 1 signal activation is overpowered in inflammatory conditions.

### Reduced Antigen-Presenting Ability of Dendritic Cells Following NICD1 Overexpression

**Figure 7:**
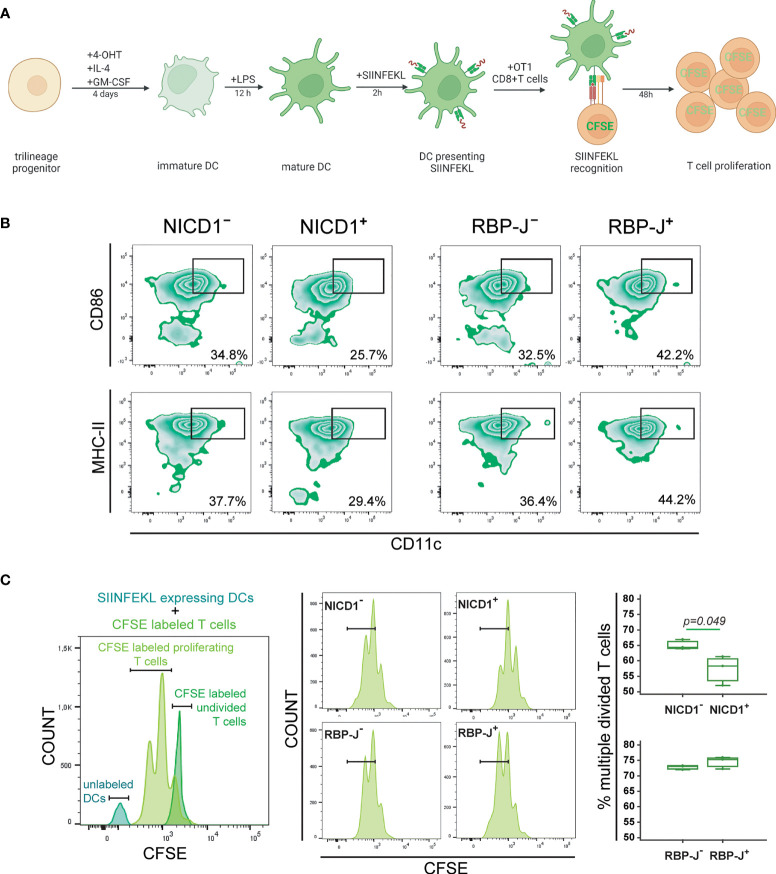
Functional characterization of dendritic cells differentiated from the common trilineage progenitor (TLP) isolated from bone marrow cells of CX3CR1CreERT2/NICD1 (NICD1) and CX3CR1CreERT2/RBP-J (RBP-J) mice. Bone marrow cells were flushed from hind limb long bones and the common TLPs were identified as CD45^+^Ly6G^−^CD3^−^B220^−^NK1.1^−^CD11b^–/lo^CD115^+^ cells using flow-cytometry. TLPs were sorted and plated under appropriate culture conditions. CD8^+^ T cells were sorted from spleens of OT-1 mice. **(A)** Experimental design of dendritic cell differentiation and functional testing. Dendritic cell differentiation was induced by the addition of interleukin 4 (IL-4) and granulocyte-macrophage colony-stimulating factor (GM-CSF). Cre-mediated recombination in *Cre*^+^ (NICD1^+^ or RBP-J^+^) littermates was induced by the addition of 4-hydroxytamoxifen (4-OHT). *Cre*^−^ (NICD1^−^ or RBP-J^−^) littermates treated with 4-OHT were used as controls. Lipopolysaccharides from *E.coli* (LPS) were added 12h before co-culture with OT-1 T cells to induce the inflammatory response. Presentation of SIINFEKL and proliferation of CFSE-labeled CD8^+^ OT-1 T cells was used as a functional assay. Created with BioRender.com **(B)** Expression of CD86 and MHCII on CD11c^+^ mature dendritic cells from NICD1 and RBP-J TLPs, stimulated by LPS. Representative dot plots showing CD86^+^CD11c^+^ and MHCII^+^CD11c^+^ subpopulations. **(C)** Quantification of divided CFSE-labeled OT-1 T cells co-cultured with SIINFEKL-expressing LPS-stimulated dendritic cells. Representative histogram showing undivided CFSE-labeled OT-1 T cells, proliferating CFSE-labeled OT-1 T cells and unlabeled dendritic cells (DCs) (*left*). Representative histograms of proliferating CFSE-labeled OT-1 T cells from *Cre*^+^ and *Cre*^−^ littermates (*middle*). Percentage of divided CFSE-labeled OT-1 T cells co-cultured with SIINFEKL-expressing LPS-stimulated dendritic cells (*right*). Individual values are presented (n=3); horizontal lines represent the median, boxes represent the interquartile range (IQR), whiskers represent 1.5 times the IQR. Statistically significant difference was determined at p<0.05, Mann–Whitney U-test.

As dendritic cells are professional antigen-presenting cells, we tested what effect Notch modulation has on their function of antigen intake, processing and presentation in the context of MHC class I molecules. Common trilineage progenitor cells from CX3CR1CreERT2/NICD1 and CX3CR1CreERT2/RBP-J mice were stimulated by IL-4 and GM-CSF, and treated with 4-OHT to induce Cre-mediated recombination. Differentiated and LPS-stimulated dendritic cells were incubated with SIINFEKL, a peptide derived from ovalbumin, and recognized specifically by transgenic T cell receptors of OT-1 mice (**Figure 7A**). The functional ability of antigen cross-presentation by dendritic cells was quantified by measuring the proliferation of sorted splenic CD8^+^ CFSE-labeled OT-1 T cells after antigen stimulation. T cell proliferation was analyzed by measuring CFSE fluorescence in daughter cells, which halves after each cell division.

We previously showed that Notch modulation did not affect the activation phenotype in maturing dendritic cells (see **Figures 3**, **4**). However, in the functional assay in which dendritic cells were matured for an additional two days following LPS-stimulation and SIINFEKL-incubation, activation markers CD86 and MHC-II were suppressed in NICD1^+^ and induced in RBP-J^+^ cells (**Figure 7B**). For testing cross-presentation, unlabeled dendritic cells were co-cultured with CFSE-labeled CD8^+^ OT-1 T cells to induce their proliferation. The percentage of divided cells was expressed compared to CFSE-labeled undivided CD8^+^ OT-1 T cells (**Figure 7C**, *left*). We observed that Notch 1 signal activation in antigen-presenting dendritic cells results in a significantly reduced proliferation rate of T cells, seen as a lower fraction of divided cells at the examined time-point, whereas Notch signaling inhibition did not have significant effect (**Figure 7C**, *right*). The results indicate that Notch 1 signaling negatively regulates dendritic cell functional cross-presenting activity.

## Discussion

The focus of our study was to characterize a bone marrow myeloid trilineage progenitor (also named MODP) of the adult mouse, with the ability to differentiate into three distinct myeloid lineages, namely osteoclasts, macrophages and dendritic cells, in the context of Notch signal modulation. The novelty of our approach is determination of the cell autonomous response to Notch signaling at the stage of the isolated trilineage progenitor. The major finding was significant induction of osteoclast differentiation and activity by suppression of canonical Notch signaling, especially under inflammatory conditions. In addition, phagocytosis by macrophages was induced upon Notch signaling inhibition, but inflammatory LPS stimuli enhanced the function of Notch 1 overexpressing macrophages as well. On the other hand, antigen presentation by LPS-stimulated dendritic cells was significantly suppressed in case of Notch 1 signal activation. These pleiotropic myeloid lineages are important effectors of homeostasis, inflammation and immune response in peripheral tissues (5), and their therapeutic modulation has major implications for infectious diseases, tolerance induction, inflammation, tumor immunotherapy and tissue repair, so there is a reasonable need to explore further their regulation by the Notch pathway.

The mononuclear phagocyte system is a family of cells including progenitors, circulating blood monocytes, resident tissue macrophages and dendritic cells as well as bone-associated osteoclasts (12, 48). Even during adulthood, myeloid progenitors of hematopoietic origin retain a certain degree of plasticity and mobility, and can be recovered from medullary and extramedullary sites, such as the spleen, liver, muscle, lungs and intestine (49). We showed that in the adult mouse bone marrow, the common trilineage progenitor can be defined as the CD45^+^Ly6G^−^CD3^−^B220^−^NK1.1^−^CD11b^–/lo^CD115^+^ subset, comprising around 5% of total hematopoietic cells. This is in line with previous studies attempting to define the trilineage progenitor (2, 4, 15, 16, 18, 19). Collectively, they showed that the progenitor is contained within the hematopoietic CD45^+^ population of non-lymphoid cells, expressing various degrees of myeloid markers, including CD115, CD11b, Ly6C, CCR2 and CX3CR1. A recent study by Grabert et al. indicated that proliferation, differentiation and survival of cells belonging to the mononuclear phagocyte system depends on signals from the M-CSF receptor (CD115) (22). We observed that virtually the entire population of CD115^+^ bone marrow progenitors expresses the chemokine receptor CX3CR1 (fractalkine receptor), justifying the use of CX3CR1CreERT2 mice to modulate Notch signaling. Other groups also confirmed that myeloid progenitors express CX3CR1, which may serve as a chemoattractant and adhesion molecule, and mediate both myeloid progenitor bone marrow recruitment as well as attraction to the site of inflammation (2, 20, 21, 50–53).

A better understanding of signaling pathways important for fate determination of the myeloid progenitor may provide novel insights into the mechanisms that control the kinetic profile and functional activity of differentiated cells (23). Since the isolated trilineage progenitor used in our study expresses substantial levels of Notch receptors, specifically Notch 1 and Notch 2, we assumed that it would be responsive to Notch signaling modulation. By using CX3CR1-driven Cre-mediated recombination, we were able to induce Notch 1 signal activation (54) or canonical Notch pathway inhibition (55) during *in vitro* differentiation of the bone marrow trilineage progenitor into macrophages, osteoclasts and dendritic cells. Notch modulation could be potentially used to manipulate myeloid lineage commitment with subsequent clinical benefits, such as modulating the development of dendritic cells to suppress autoreactive T cells, macrophages to mediate tissue repair or osteoclasts to remodel the hematopoietic niche (5, 7, 8, 10). Moreover, dysregulation of Notch signaling was associated with pathological processes, from different types of cancer to immune disorders. Therapeutic strategies targeting Notch signaling have been developed, and while complete inhibition of Notch signaling induces severe and intolerable side effects, targeting individual Notch receptors may prove beneficial. Therapeutic Notch inhibition, although primarily used in cancer treatment, is now increasingly tested in inflammatory disorders (56). Several factors need to be considered in designing therapies directed towards myeloid cells, including the targeted pathway and tissue context. Progenitor plasticity and diversity of cell subsets that can be derived from the same ancestry may undermine attempts to develop successful approaches (5).

Macrophages express both Notch receptors and ligands (57), and Notch signaling was shown to have a critical role in macrophages during inflammation and infection (58). Furthermore, there is a bidirectional regulation of TLRs and Notch pathway, with the ability of TLR4 (targeting LPS) to enhance Notch signaling effect (59–62). We showed that *in vitro* Notch modulation in basal conditions did not affect the number of macrophages differentiated from the trilineage progenitor by M-CSF. Nevertheless, we observed increased *Il12b* transcript levels in RBP-J deficient macrophages, similar to Krishnasamy et al. (63). The suppressive effect of Notch signaling seems to be mediated by Hes1 (64), highly upregulated in differentiating macrophages. In contrast, Xu et al. showed the dependence of *Il12b* induction on RBP-J expression, but the study was conducted on LPS-stimulated bone marrow-derived macrophages from the Rbpj^fl/fl^Mx1-Cre mouse strain after *in vivo* deletion (65). In experiments that mimic inflammatory conditions by addition of LPS, induction of activation markers (CD64 and CD86) was similar in NICD1^+^ and NICD1^–^ macrophages, indicating that NICD1 does not propagate an inhibitory signal. This phenotype is consistent with the finding of increased Notch-driven M1 macrophage polarization (65–67). Phagocytic activity was also enhanced in NICD1^+^ macrophages upon LPS treatment, although it was suppressed in basal conditions. There are many reported findings of Notch activation mediating the macrophage proinflammatory response and both *in vitro* and *in vivo* Notch signaling inhibition diminishing inflammation (59, 61, 67–69). Contrary evidence showed that RBP-J signaling induces M2 macrophage polarization (70) and NICD1/NICD2 overexpression reduces the TLR4-triggered proinflammatory response (71). We observed expression of macrophage activation markers as well as increased phagocytosis upon RBP-J signaling inhibition under inflammatory stimulation. This is in line with the finding that RBP-J deletion impairs macrophage maturation from Ly6C^hi^ monocytes, leading to higher proliferation rates and increased inflammatory profile (63). The apparent discrepancy possibly indicates that the inflammatory stimulus by LPS acts upstream of RBP-J and is able to override the effect of Notch modulation on macrophage differentiation. This is in turn consistent with the finding that only around 10% of TLR4-inducible genes were partially dependent on RBP-J (65).

There is sufficient data suggesting the importance of Notch signaling in regulation of dendritic cell differentiation and function, however the nature of the regulation is still elusive (72, 73). Moreover, the dendritic cell lineage comprises a number of subsets (such as classical or conventional subset 1 and 2, inflammatory or monocyte-derived, plasmacytoid, Langerhans) that may respond differently to modulation of the canonical Notch – RBP-J pathway (9, 74). A common method for studying dendritic cell biology, applied in our study, is *in vitro* stimulation by GM-CSF and IL-4. This culture system mostly induces inflammatory dendritic cells (which share similarities with macrophages and conventional dendritic cell subset 2), expressing high levels of MHC-II, costimulatory molecules and CD11c (9), congruent with our findings. In our *in vitro* setting, RBP-J deletion in the trilineage progenitor did not affect dendritic cell proliferation nor antigen presentation, but did enhance costimulatory molecule expression. A study by Cheng et al. found that early progenitors (Notch-1–null embryonic stem cells or Notch-1–deficient hematopoietic lineage-negative bone marrow progenitors) had a reduced capacity to differentiate into dendritic cells (75). However, activation of Notch signaling by fibroblasts expressing JAG 1 resulted in the accumulation of immature dendritic cells, whereas withdrawal of Notch signaling permitted their differentiation. Conventional dendritic cells seem to be more dependent on Notch signaling than the inflammatory subset, since stimulation with DLL1 optimizes their *in vitro* generation and T cell cross-priming (74). Moreover, conventional dendritic cell terminal differentiation, migration and antigen cross-presentation were decreased by impaired Notch 2 signaling (76), and their subsets were reduced by Notch 2 signal inhibition or RBP-J inactivation *in vivo* (77, 78). LPS induces maturation of dendritic cells, characterized by a high T cell-stimulatory function and poor antigen uptake ability (79). We showed that LPS-stimulated NICD1^+^ dendritic cells possess reduced CD8^+^ T cell cross-priming ability, paralleled by downregulation of activation markers (MHC-II and CD86). Our results may point to an overall modest role of Notch signaling in differentiation of monocyte-derived dendritic cells and negative regulation of T-cell priming by mature dendritic cells through Notch 1.

Recent work demonstrated distinct actions of each Notch receptor in bone remodeling and osteoclastogenesis under physiological conditions and during inflammation (29). We showed that Notch 1 signal activation in differentiating osteoclasts reduces the number of bone resorbing TRAP^+^ osteoclasts, and the suppression effect is still clearly visible under LPS stimulation. The work by Bai et al., as well as Sekine et al. confirms the inhibitory role of Notch 1 in differentiation of osteoclasts, by using osteoclast progenitors from Notch 1 conditional knockout mice and activating Notch 1 antibodies, respectively (80, 81). In the *in vivo* models (with loss or gain of function for Notch receptors) driven by osteoclast, osteoblast or osteocyte lineage promotors (29, 82), Notch 1 signal activation inhibited both osteoblast and osteoclast differentiation, and caused an osteopetrotic phenotype (80, 83–85). In addition, RBP-J seems dispensable for the function of osteoblasts, osteocytes and osteoclasts under basal conditions, considering that the inactivation of RBP-J in these cell lineages *in vivo* did not produce a skeletal phenotype (86, 87). By culturing bone marrow cells on immobilized Notch ligands, Ashley et al. showed that Notch signaling suppresses osteoclast differentiation from non-adherent (immature), whereas it enhances osteoclast maturation from adherent (more committed) bone marrow-derived macrophages (41). Accordingly, we confirmed the inhibiting effect of JAG 1 on trilineage progenitor differentiation, and reversal of inhibition by using neutralizing anti-Notch 1 antibodies. Moreover, NICD1 overexpression exhibited effects on the molecular level, by downregulating osteoclast differentiation genes *Fos* and *Ctsk*, and, in inflammatory conditions, by halving RANK expression on differentiating osteoclasts. However, we did not study the effect of Notch 2 signal activation, reported to stimulate osteoclast differentiation by direct and indirect mechanisms (29). Nonetheless, osteoclast inhibition by NICD1 overexpression and stimulation by RBP-J deletion could mean that either Notch 1 is more important at the stage of trilineage progenitor or that Notch 2 has important effects through non-canonical signaling pathways.

In numerous inflammatory disorders, osteoclastogenesis is stimulated, mainly through direct or indirect effects of proinflammatory cytokines on osteoclast progenitors in a RANKL-dependent or -independent manner (88, 89). LPS modulates osteoclastogenesis by its direct effect on osteoclast progenitors, with the ability to suppress differentiation of RANKL-naïve progenitors and enhance differentiation of RANKL-primed progenitors, in part mediated by TNF (46, 47). Our finding underlines the essential role of signaling through Notch 1 in regulation of osteoclastogenesis, which overpowers the inflammatory LPS stimulus. Furthermore, by inhibiting downstream Notch signaling in the trilineage progenitor, we showed increased osteoclastogenesis, with a specific increase in the number of large TRAP^+^ bone resorbing cells, upregulation of *Ctsk* gene and stimulation of RANK protein expression. The effect of RBP-J deletion was augmented further with LPS stimulation. Zhao et al. also addressed this important role of Notch signaling in inflammation, by showing that RBP-J limits TNF-induced osteoclastogenesis, while TNF stimulation greatly induces osteoclastogenesis in RBP-J deficient osteoclast progenitors (86). Together with our results, this confirms the role of RBP-J as a negative regulator of osteoclastogenesis, especially in the inflammatory setting, functioning by balancing osteoclastogenic activators and repressors.

## Conclusions

**Figure 8:**
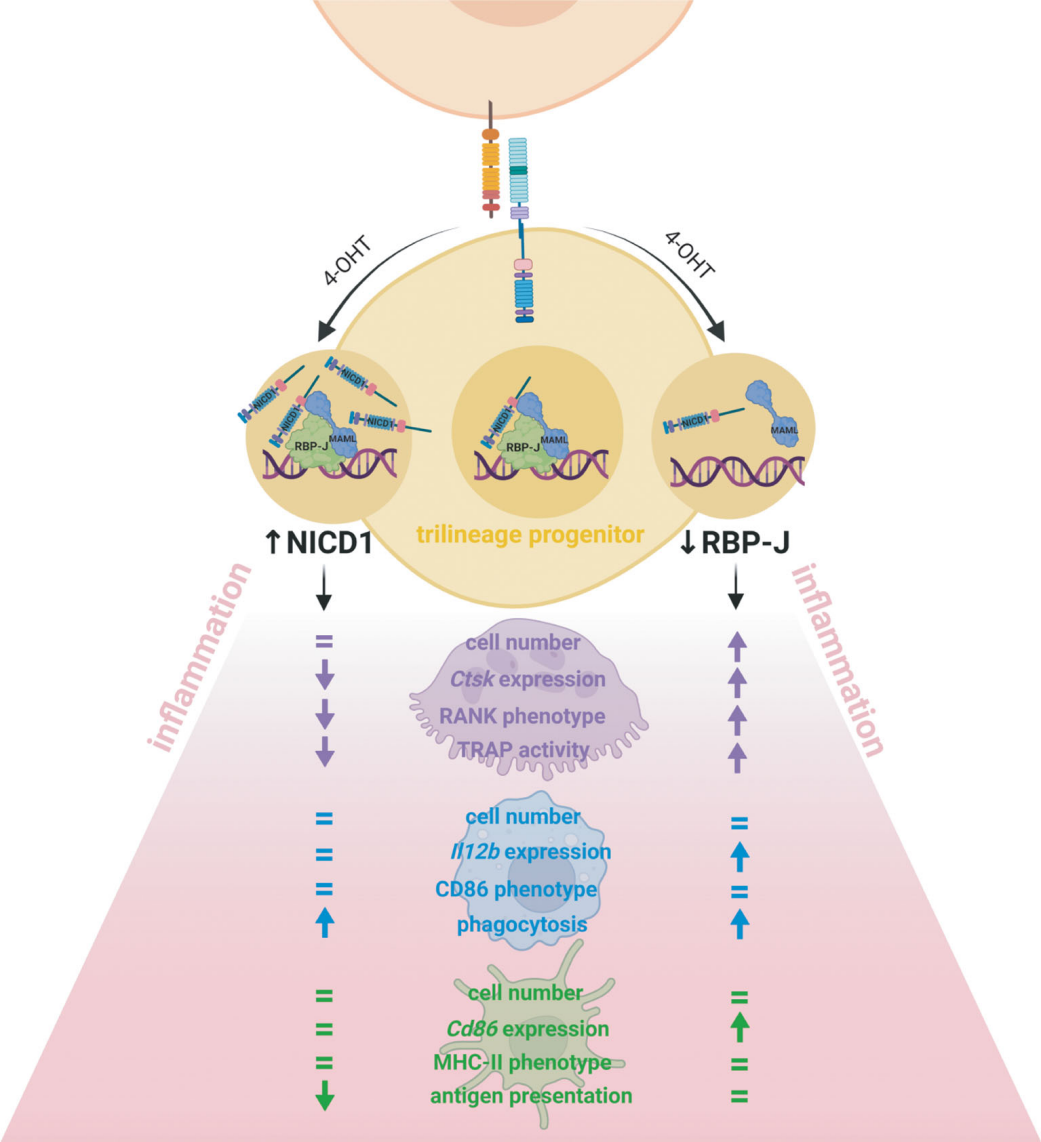
Effect of Notch signal modulation on the bone marrow trilineage progenitor (TLP) in the inflammatory context. Conditional inducible transgenic mouse strain in which expression of the Cre recombinase is driven by the monocyte marker CX3CR1 was crossed with either a mouse strain in which Notch receptor 1 intracellular domain (NICD1) is overexpressed or a mouse strain in which the transcriptional factor RBP-J is deleted upon Cre-mediated recombination. The transgene was activated *in vitro*, by 4-hydroxytamoxifen (4-OHT) treatment of isolated TLPs, from the beginning of differentiation. The effects of Notch modulation were assessed by cell proliferation, differentiation gene expression, LPS-stimulated activation phenotype and functional assays, and the major findings are schematically presented. The clearest effect was observed in cells of the osteoclast lineage. Deletion of RBP-J involved in Notch canonical signaling enhanced osteoclast progenitor proliferation and osteoclast *Ctsk* differentiation gene expression as well as RANK abundance and TRAP activity, whereas NICD1 overexpression had the opposite effects. The macrophage lineage responded to RBP-J deletion by upregulation of *Il12b* differentiation gene expression, without significant difference in the activation phenotype. However, both NICD1 overexpression and RBP-J deletion enhanced LPS-induced phagocytosis, indicating that other pathways beside Notch critically regulate macrophage activity. The Notch pathway seems to be less active during dendritic cell commitment from TLP, so Notch modulation did not affect dendritic cell proliferation and their activation phenotype but had a moderate effect on *Cd86* differentiation gene expression. However, in the functional assay, mature dendritic cells overexpressing NICD1 showed diminished activation markers and a reduced antigen-presenting ability.

Incessant myeloid progenitor trafficking continuously replenishes bone marrow cavities and, in addition, surveils extramedullary sites. In peripheral tissues, progenitors sense pathogens or damaged cells through pattern recognition receptors and react to inflammatory stimuli by differentiating into mature myeloid cells, as well as by secreting cytokines, thus participating in tissue homeostasis and immune responses. Our study design allowed us to interpret the effect of Notch modulation from the stage of the myeloid trilinege progenitor and its intrinsic role in lineage polarization and activity, which has not been studied previously (**Figure 8**). While the major finding of our study indicates a negative role of Notch – RBP-J canonical pathway in the *in vitro* trilineage differentiation, especially in the osteoclast lineage, many authors proved the importance of Notch signaling and even differentiation enhancement by Notch activation. The discrepancies between findings emphasize that Notch signaling is heavily cell-, ligand/receptor- and context-dependent and influenced by the experimental setting. Since present-day therapeutic approaches are focused primarily on inhibition of Notch signaling, these effects warrant additional attention, as they may bring about unwanted activating responses in macrophages and osteoclasts.

## Data Availability Statement

The raw data supporting the conclusions of this article will be made available by the authors, without undue reservation.

## Ethics Statement

The animal study was reviewed and approved by Ministarstvo poljoprivrede (Ministry of Agriculture), Uprava za veterinarstvo i sigurnost hrane, Zagreb, Croatia and the Ethics Committee of the University of Zagreb School of Medicine, Zagreb, Croatia.

## Author Contributions

MF, DG, DF and AŠ designed the study. MF, DG, DF, AŠ, DŠ, TK and IK performed the experiments. MF, DG, DF, AŠ, DŠ, TK, MA and NK acquired and analyzed data. MF, DG, DF, AŠ, NK, IK and MA interpreted the results. MF, DG, DF and AŠ prepared the manuscript. All authors critically revised the manuscript and approved the final version. All authors contributed to the article and approved the submitted version.

## Funding

This work was supported by grants from the Croatian Science Foundation (IP-2018-01-2414, DOK-2018-09-4276, UIP-2017-05-1965 and IP-2020-02-2431) and by Scientific Center of Excellence for Reproductive and Regenerative Medicine, Republic of Croatia, and by the European Union through the European Regional Development Fund, under grant agreement No. KK.01.1.1.01.0008, project “Reproductive and Regenerative Medicine - Exploring New Platforms and Potentials”.

## Conflict of Interest

The authors declare that the research was conducted in the absence of any commercial or financial relationships that could be construed as a potential conflict of interest.

## Publisher’s Note

All claims expressed in this article are solely those of the authors and do not necessarily represent those of their affiliated organizations, or those of the publisher, the editors and the reviewers. Any product that may be evaluated in this article, or claim that may be made by its manufacturer, is not guaranteed or endorsed by the publisher.
