# Supplementary figures and images for: Inhibition of Notch Signaling Stimulates Osteoclastogenesis From the Common Trilineage Progenitor Under Inflammatory Conditions
Source: Front Immunol. 2022 Jul 5;13:902947. doi: 10.3389/fimmu.2022.902947 (PMC9294223; doi:10.3389/fimmu.2022.902947)

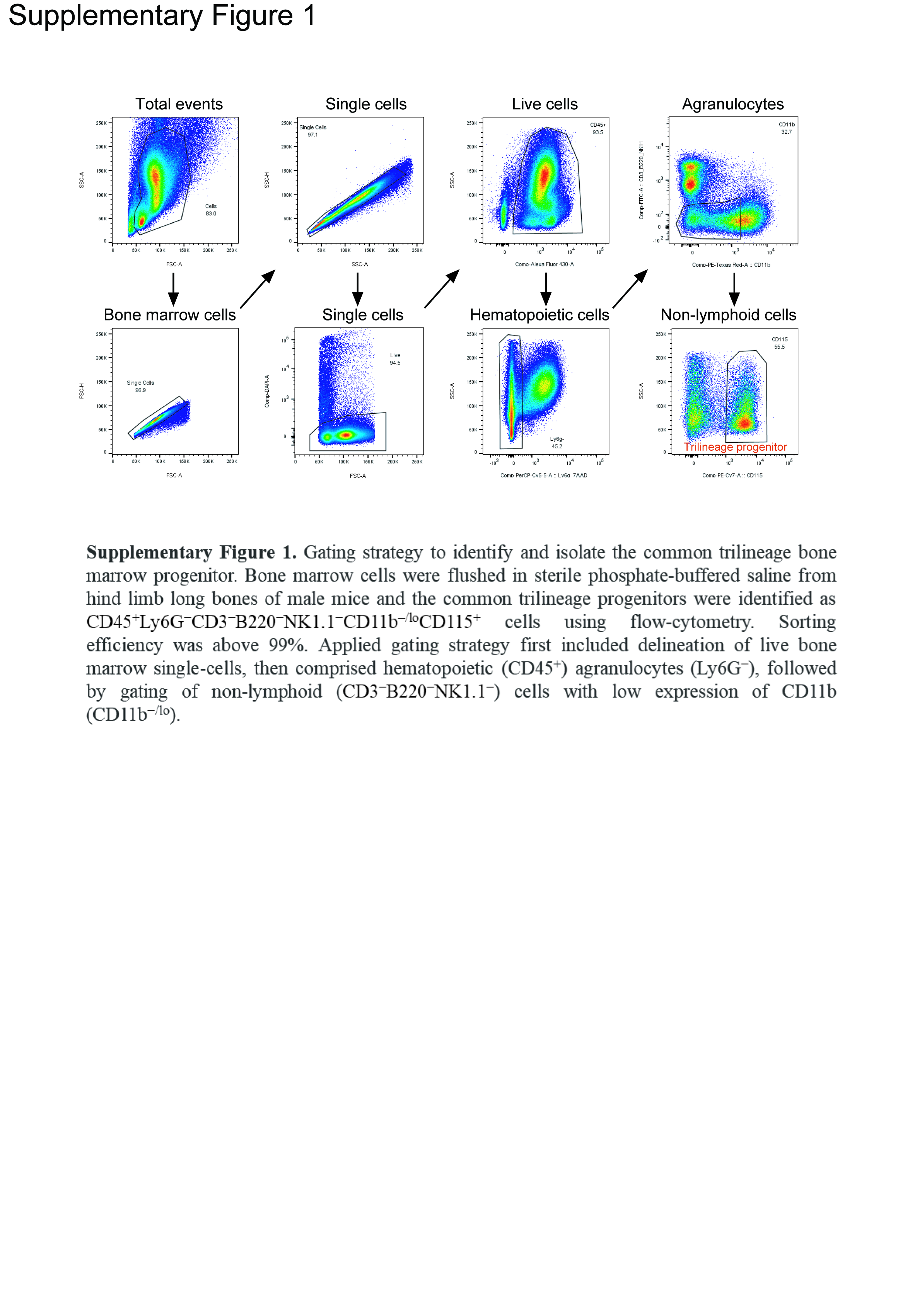

Supplement: Supplementary file 1 [file Image_1.tif]

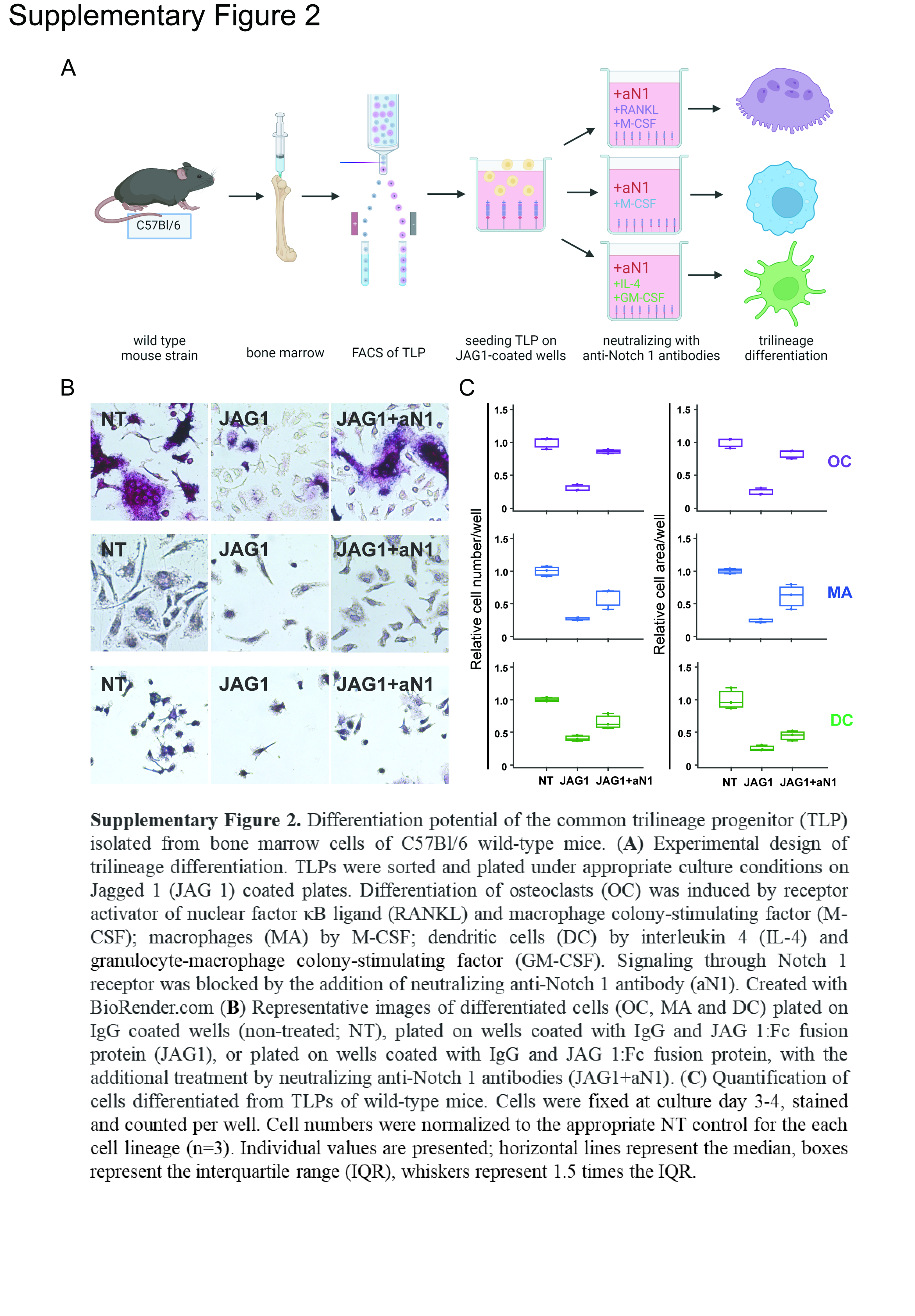

Supplement: Supplementary file 2 [file Image_2.tif]

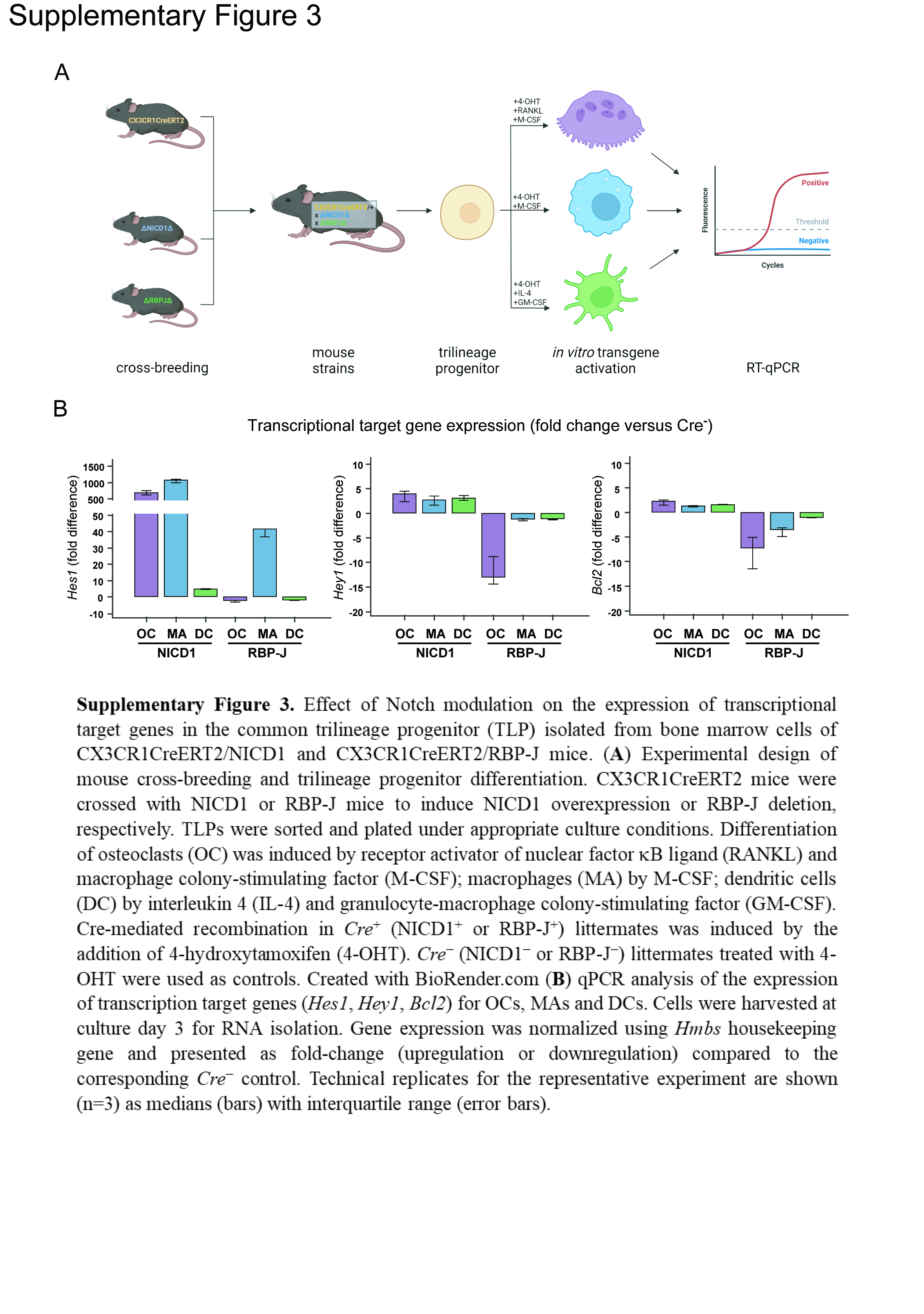

Supplement: Supplementary file 3 [file Image_3.tif]

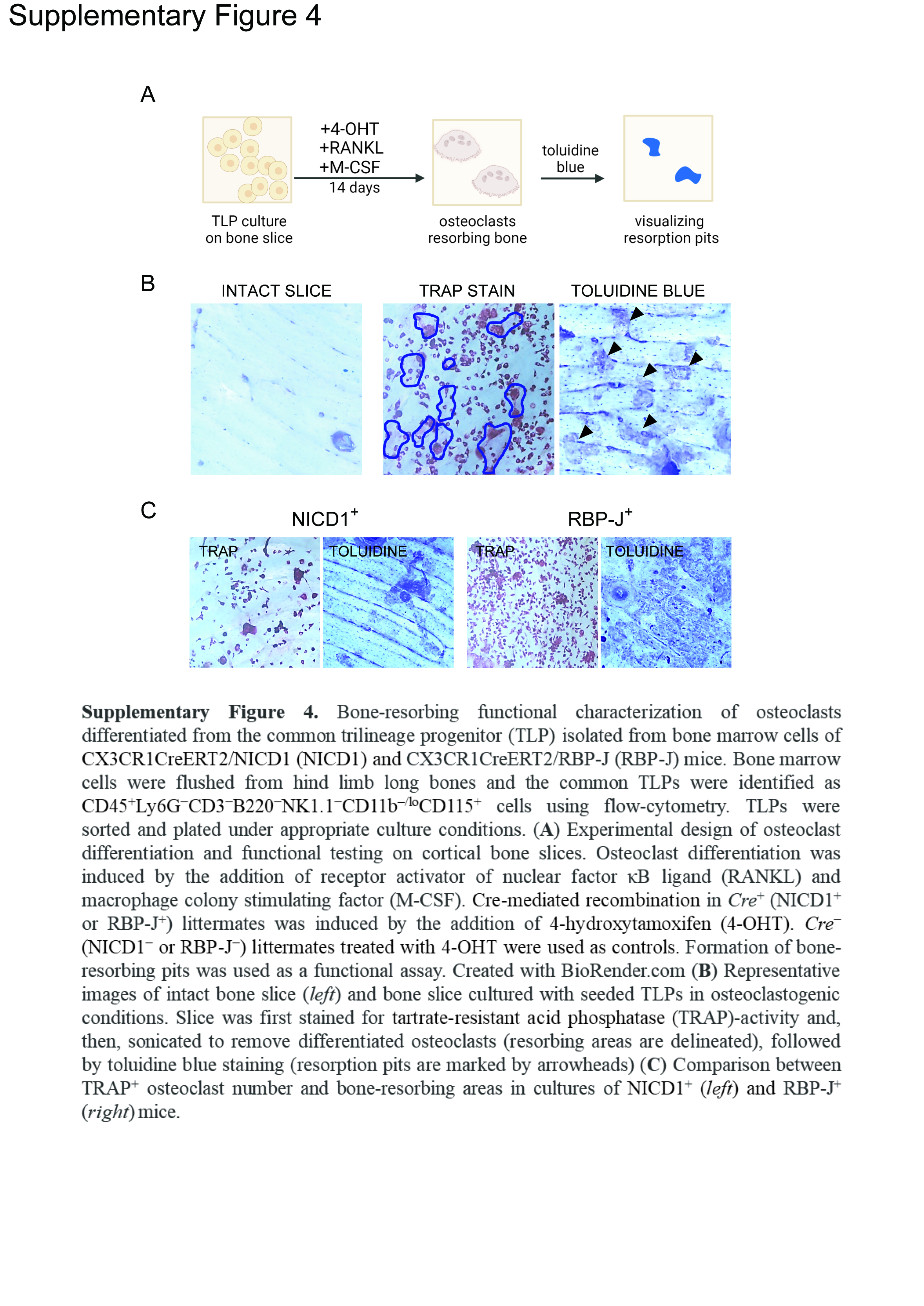

Supplement: Supplementary file 4 [file Image_4.tif]
